# Men’s Perceptions and Emotional Responses to Becoming a Caregiver Father: The Role of Individual Differences in Masculine Honor Ideals and Reputation Concerns

**DOI:** 10.3389/fpsyg.2019.01442

**Published:** 2019-06-28

**Authors:** Pelin Gul, Ayse K. Uskul

**Affiliations:** ^1^School of Psychology, University of Kent, Canterbury, United Kingdom; ^2^Department of Psychology, Iowa State University, Ames, IA, United States

**Keywords:** gender roles, caregiver, breadwinner, masculine honor, precarious manhood, reputation concerns, emotion perception, stereotype content model

## Abstract

Despite the rising number of men and women in counter-stereotypical roles, it is rare for men to serve as primary caregivers in families with dependent children. In two studies, we examined how British men and women perceive and emotionally react to primary caregiver (vs. breadwinner) fathers, whether their perceptions and reactions are contingent upon individual differences in masculine honor endorsement, and the potential mediators in these relationships. Results showed that both men and women perceived the primary caregiver fathers more positively – warmer and not less competent – than the primary breadwinner fathers, yet endorsement of masculine honor ideals increased men’s (but not women’s) tendency to attribute less positive emotions (e.g., proud and satisfied) and more negative emotions (e.g., ashamed and resentful) to the primary caregiver (vs. breadwinner) fathers. Mediated moderation analyses showed that for men with high masculine honor orientation, their less positive and more negative emotional attributions were driven by perceived loss of reputation among male friends, whereas for men with low masculine honor orientation, their more positive and less negative emotional attributions were driven by perceived gain of wife’s and children’s admiration. By shifting the focus to men’s individual differences and motives, these findings offer nuanced explanations for why some men may feel eager about serving as caregiver fathers, whereas others may feel reluctant to do so.

## Introduction

Radical changes in the social, economic, and scientific domains since the beginning of the 20th century have contributed to the advancement of women’s status in society, mainly through women’s increased level of educational attainment and labor force participation (England, 2010). Today women are present in all industries which were once filled by only men, including business, politics, science, and technology, and compete with men for leadership positions (England, 2010; McGuinness, 2018). It has increasingly become the norm for women in developed societies to be employed, even when they are mothers (Cory and Stirling, 2015). Survey data show that across Europe, 31% of mothers with dependent children are breadwinners, bringing in at least half of the household income (Cory and Stirling, 2015). In the United Kingdom, where the current studies were conducted, the percentage of maternal breadwinners increased from 23% in 1996 to 33% in 2013 (Cory and Stirling, 2015). Reflecting these changes in earning structures, attitude surveys show that British men’s and women’s support for a traditional family division of labor has been declining since the mid-1980s (Braun and Scott, 2009; Park et al., 2013).

Nevertheless, women’s increased labor force participation continues to pose considerable work-family challenges, especially to mothers (Park et al., 2013; Cory and Stirling, 2015). Even though men are becoming increasingly involved in household chores and childcare tasks, it is still very rare for men to become stay-at-home fathers in couple families with dependent children (Bianchi and Milkie, 2010; Connolly et al., 2016). Women report doing a disproportionately greater amount of domestic work and take on more childcare responsibilities than men, even when they work full-time (Hochschild and Machung, 1989; Bianchi and Milkie, 2010; Park et al., 2013). For example, in the United Kingdom, surveys show that more than half of the women view their contribution to housework and childcare as being unfair, whereas almost half of the men report contributing less than their fair share (Park et al., 2013).

There are many benefits to men’s involvement in childcare. Studies have shown that greater paternal caregiving is related to greater marital satisfaction (Kluwer et al., 1996), feelings of competence as a father (Baruch and Barnett, 1986), increased social bonding with one’s children, and enhanced self-esteem and well-being of one’s children (Deutsch et al., 2001; Goncy and Van Dulmen, 2010, also see Pleck, 2007). Moreover, some researchers argue that men’s involvement in housework and childcare is the key solution to achieving work-life balance and greater gender equality in the division of domestic roles (Deutsch et al., 1993; Jackson, 2006; Croft et al., 2015). Given these benefits of paternal caregiving, along with the views that traditional gender roles are becoming less optimal for successful functioning of families (Ickes, 1993), it is important to understand the psychological factors that prevent men from taking on caregiver roles, as well as those that encourage men to do so.

Despite the societal and institutional pressures for change in traditional gender roles and increasing societal acceptance of men and women occupying non-normative roles (Coleman and Franiuk, 2011; Park et al., 2013), psychological processes can still play a role in inhibiting men from participating in childcare. In their extensive review on the psychological mechanisms that inhibit men’s interest in communal roles, Croft et al. (2015) suggest one important factor to be negative judgments of others which may threaten men’s masculinity. Inspired by this conclusion, we designed the present study to examine how British men and women currently perceive and emotionally respond to male targets in primary caregiver (vs. breadwinner) roles, how men respond emotionally to the possibility of becoming a primary caregiver (vs. breadwinner) father themselves and whether their perceptions and emotional responses are contingent upon their beliefs about the importance of masculine reputation.

### Social Judgments of Caregiver and Breadwinner Fathers

In the current study, social judgments of male targets who serve as a primary caregiver (vs. breadwinner) role within their marriage were examined with regard to attribution of warmth-related and competence-related traits as well as emotional attributions. Studies conducted with diverse United States samples and across many cultural groups have consistently revealed that warmth (comprising traits such as morality, sincerity, and friendliness) and competence (comprising traits such as efficacy, skill, and intelligence) emerge as the two fundamental dimensions of social judgment (stereotype content model; Fiske et al., 2007; Cuddy et al., 2008, 2009). According to the stereotype content model, warmth and competence stereotypes, respectively, stem from the perceived competitiveness and social status of the individuals or groups, and lead to distinct interpersonal emotions such as admiration, respect, contempt, envy, and pity (Cuddy et al., 2008). Individuals perceived as rivals, competitors, harmful, or threatening are stereotyped as lacking warmth, whereas those perceived as cooperators are stereotyped as warm. Individuals perceived as high in status (i.e., those in prestigious positions and jobs, and who are resourceful and economically successful) are stereotyped as competent, whereas those low in status are stereotyped as lacking competence. Therefore, given the lower status of caregiving roles on the one hand (Ridgeway and Correll, 2004) and the communality-related traits that the caregiving roles demand on the other hand (Eagly and Steffen, 1984), one would expect the caregiver targets to be attributed a higher level of warmth and a lower level of competence compared with the breadwinner targets (see Eckes, 2002; Gaunt, 2013 for a similar prediction).

A number of studies that have addressed people’s perceptions of warmth-related and competence-related traits of married men who take on the primary caregiver and breadwinner roles revealed findings that are generally in line with the stereotype content model’s predictions. For example, Gaunt (2013) found that primary caregiver fathers were perceived as warmer but less competent than primary breadwinner fathers. Similarly, Coleman and Franiuk (2011) found that men who take paternity leave are perceived as warmer and more moral, but less competent than men who continue working full-time after the birth of a child. Another study examining perceptions of men who seek flexible work arrangements to help with childcare at home versus those who continue to work full-time revealed similar findings with regards to perceived warmth, but the men who took flexible work arrangements were not rated as less competent (Vandello et al., 2013). Similar to these previous findings, we predicted that caregiver fathers will be perceived as warmer, but not more competent than breadwinner fathers (Hypothesis 1).

### Endorsement of Masculine Honor Ideals as a Moderator

In addition to the fundamental judgments of warmth and competence, emotional reactions can also reveal prejudice toward targets in non-traditional roles. In fact, research shows that negative emotional reactions are more effective in motivating prejudice toward men and women in non-traditional roles than judgments of warmth and competence (e.g., Stangor et al., 1991; Cuddy et al., 2007). Related to this point, several studies have found that despite rating caregiver fathers as warmer, people hold more unfavorable attitudes and react with more negative emotions to primary caregiver fathers than to primary breadwinner fathers (Riggs, 1997; Brescoll and Uhlmann, 2005; Coleman and Franiuk, 2011).

Importantly, negative evaluations of male caregivers depend on the characteristics of the perceiver (e.g., Gaunt, 2013; also see Eagly and Karau, 2002; Hoyt, 2012 for evaluations of targets in other types of non-traditional roles). For instance, Gaunt (2013) found that traditional gender ideologies moderate social judgments of primary caregiver and breadwinner fathers, whereby individuals with stronger (vs. weaker) traditional gender ideologies attributed less positive emotions and more negative emotions to a caregiver father. Here we propose that another relevant perceiver characteristic which may moderate perceivers’, and especially male perceivers’, emotional attributions to caregiving is the individuals’ endorsement of masculine honor ideals (Barnes et al., 2012; Saucier and McManus, 2014; Saucier et al., 2016). It is argued that men who adhere to masculine honor ideals are more receptive to potential cues and situations that may threaten their masculine reputation, respond to reputation threats more negatively, and engage in more stereotypically masculine behaviors to protect and maintain their masculine image (Saucier and McManus, 2014). For instance, studies have shown that American men’s endorsement of masculine honor ideals was related to reacting more angrily and aggressively to insults or provocations (Cohen et al., 1996; Saucier et al., 2016), striving to be more muscular (Saucier et al., 2018), and perceiving a man who walks away from personal threats as less manly (O’Dea et al., 2017). Our own studies revealed that British men’s endorsement of masculine honor ideals was related to higher interest in stereotypically masculine occupations, college majors, and leisure activities, and lower interest in stereotypically feminine ones (Gul and Uskul, 2019).

Moreover, when men experience threats to their masculine reputation, they become more avoidant of feminine expressions and preferences (Bosson et al., 2005; Cheryan et al., 2015), less supportive of paternal care of children (Kosakowska-Berezecka et al., 2016), and less willing to seek flexible working hours for childcare reasons (Vandello et al., 2013). By the same token, we suggest that serving as a caregiver may be taken by some men as a threat to their masculine reputation, especially if it compromises their breadwinning role, given that the ability to financially provide for one’s family is a significant part of men’s gender role (Gilmore, 1990; Vandello and Bosson, 2013). Therefore, men may attribute negative emotions to primary caregiver fathers or to the idea of becoming a primary caregiver father, especially if they strongly endorse masculine honor ideals. Specifically, we predicted that men’s endorsement of masculine honor ideals will increase their tendency to attribute *less* positive emotions (e.g., satisfaction and proud) and *more* negative emotions (e.g., shame and humiliation) to primary caregiver or to the idea of becoming a primary caregiver father themselves (Hypothesis 2).

Although our Hypothesis 2 applied only to male participants, few studies have shown that women can also endorse masculine honor ideals and hold men to these ideals, even though women’s endorsement of masculine honor ideals does not reflect their personal concern with maintaining a masculine reputation as it does with men’s (e.g., Barnes et al., 2012). Therefore, we also explored whether women’s emotional attributions to caregiver (vs. breadwinner) fathers are contingent upon their endorsement of masculine honor ideals, primarily to find out whether the moderating role of masculine honor ideals is unique to men.

### Reputation Concerns as Mediators

Numerous studies show that when men take on communal roles, they risk negative evaluations and loss of masculine reputation. However, these negative evaluations are often expressed by status-relevant observers such as other men or work colleagues (e.g., Laroche and Livneh, 1983; Bosson et al., 2006), or appear in status-relevant domains such as work settings (e.g., Moss-Racusin et al., 2010). In contrast, other studies show that men who contribute to childcare tasks are judged positively, particularly by their wives and family (Hochschild and Machung, 1989; Deutsch and Saxon, 1998). For example, in a study conducted with dual-earner couples with children, Deutsch et al. (2003) found that women were more grateful to their husbands when they contributed a greater percentage of parenting, and men felt more appreciated as a parent when they did relatively more than their wives did. Thus, it is reasonable to assume that a man who contributes to childcare tasks may intuit that he is gaining his wife’s praise and admiration, despite also worrying about losing reputation in the eyes of other men for compromising his provider role.

Moreover, we suggest that men’s perceptions of what their wives, children, and male friends would think of them if they were a primary caregiver or a breadwinner may depend on their endorsement of masculine honor ideals. These perceptions may in turn explain high and low masculine honor-oriented men’s emotional attributions to the idea of becoming a caregiver (vs. breadwinner). More specifically, men’s higher endorsement of masculine honor ideals may be linked to perceiving higher reputation loss in the eyes of other men as a result of becoming a primary caregiver (vs. breadwinner). Perceived loss of reputation in the eyes of other men may in turn explain higher masculine honor-oriented men’s negative emotional attributions to the idea of becoming a primary caregiver (vs. breadwinner). In contrast, because men with lower masculine honor orientation are not as concerned about maintaining their masculine reputation, lower endorsement of masculine honor ideals may be linked to higher perceived admiration of one’s wife and children as a result of becoming a primary caregiver (vs. breadwinner). Higher perceived admiration from one’s wife and children may in turn explain lower masculine honor-oriented men’s positive emotional reactions to the idea of becoming a caregiver (vs. breadwinner).

**FIGURE 1:**
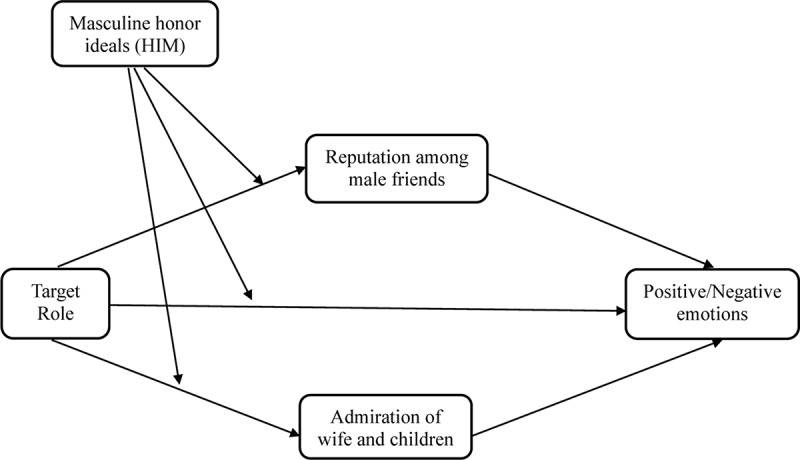
Conceptual diagram of the effect of Target Role (caregiver vs. breadwinner), Masculine honor ideals (HIM), and Target Role × HIM interaction on men’s positive and negative emotional attributions mediated by perceived reputation among male friends and perceived admiration of wife and children.

Thus, we hypothesized that men’s endorsement of masculine honor ideals would moderate their perceived reputation loss among male friends and perceived admiration from family (wife and children) if they were a primary caregiver (vs. breadwinner) (Hypothesis 3). We further hypothesized that higher masculine honor-oriented men’s *less* positive and *more* negative emotional reactions to becoming a primary caregiver (vs. breadwinner) would be mediated by their perceived loss of reputation among male friends (Hypothesis 4a), and lower masculine honor-oriented men’s *more* positive and *less* negative emotional reactions to becoming a primary caregiver (vs. breadwinner) would be mediated by perceived gain of their wife’s and children’s praise and admiration (Hypothesis 4b). Our predictions are synthesized in the mediated moderation model depicted conceptually in Figure 1.

### The Present Research

Across two studies, we examined British men’s and women’s perceptions of and emotional attributions to primary caregiver (vs. breadwinner) fathers, and whether their perceptions and emotional attributions are contingent upon endorsement of masculine honor ideals. Following methods previously used by other researchers (e.g., Riggs, 1997; Coleman and Franiuk, 2011; Gaunt, 2013; Vandello et al., 2013), we asked participants to read profiles of male targets who were either described as a caregiver father married to a breadwinner mother, or a breadwinner father married to a caregiver mother. We assessed participants’ perceptions of the male targets on core dimensions of social judgment (i.e., warmth and competence), and their emotional attributions using moral emotions, based on the logic that moral emotions reflect perceivers’ socially desirable norms and standards (Tangney et al., 2007). Study 1 recruited both men and women, and Study 2 recruited only men. In Study 2, we also assessed men’s perceptions of how becoming a caregiver (vs. breadwinner) father may reflect on the various aspects of their reputation, considering from the perspective of status-relevant observers (male friends), and status-irrelevant observers (their wife and children) in order to test the mediated moderation patterns predicted.

## Study 1

The aim of Study 1 was to examine British men and women’s perceptions of and emotional attributions to primary caregiver (vs. breadwinner) fathers, and whether their perceptions and emotional attributions are contingent upon the endorsement of masculine honor ideals.

### Methods

#### Participants

Sample size was not determined a priori; we attempted to reach approximately 150 participants following a similar previous study (Vandello et al., 2013). A total of 176 participants from the United Kingdom, recruited through Prolific Academic, completed an online survey advertised as “a study on impression formation of married couples.” Participants were a mixture of university students and working adults. Excluding 16 participants who were non-heterosexual^1^, and 6 participants who failed to pass simple attention checks left data from 155 participants used in the analyses (71 men; *M* = 33.59, *SD* = 9.40; 98% White-British). *Post hoc* power analysis conducted with G^*^Power indicated that sample size of 155 would have 0.86 power to detect the smallest effect size (β = 0.24) obtained from our significant three-way interaction effect (Target Role × Participant Gender × Masculine Honor Ideals) on the outcome variables.

Participants’ highest education levels varied (21% high-school, 21% college, 47% undergraduate, 11% postgraduate) and a majority of them were married or in a relationship (47% married, engaged or in a civil union, 23% in a dating relationship, 25% not in a relationship, 5% divorced). Participants had middle socioeconomic status (*M* = 52.85, *SD* = 19.23 assessed from 0 = low to 100 = high), slightly liberal political orientation (*M* = 62.50, *SD* = 21.52 assessed from 0 = extremely conservative to 100 = extremely liberal), and they scored low on religiosity (*M* = 1.47, *SD* = 0.82 measured from 1 = not at all to 5 = extremely). Men and women in our sample did not differ in any of the demographic characteristics except for age, *t*(153) = 2.80, *p* = 0.006. Women (*M* = 35.49, *SD* = 9.07) were slightly older than men (*M* = 31.34, *SD* = 9.35)^2^.

#### Design and Procedure

The study used a 2 × 2 × 2 design with *target role* (breadwinner vs. caregiver) and *participant gender* (men vs. women) as between-subject factors, and *masculine honor ideals* measured as a continuous moderator.

#### Profiles

Participants evaluated profiles of male targets described as either a full-time caregiver married to a breadwinner or a full-time breadwinner married to a caregiver, followed by a completion of a serious of measures related to the profiles. Participants in the *breadwinner condition* read the following target description:

Michael is 34 years old, married and a parent to 5-year old son and a 2-year old daughter. He is a successful manager in a big firm. He leaves home early in the morning and usually returns in the evening around 7 pm. His wife is a stay-at-home mom. She picks up the children from kindergarten and takes care of the housework and childcare (cooking, feeding the children, giving them a bath, doing the laundry, driving the children to social and other activities, etc.).

Participants in the *caregiver condition* read the following target description:

Michael is 34 years old, married and a parent to 5-year old son and a 2-year old daughter. He is a stay-at-home dad. He picks up the children from kindergarten and takes care of the housework and childcare (cooking, feeding the children, giving them a bath, doing the laundry, driving the children to social and other activities, etc.). His wife is a successful manager in a big firm. She leaves home early in the morning and usually returns in the evening around 7 pm.

#### Measures

##### Trait judgments

Participants rated the target on a number of traits measuring warmth/morality and competence taken after Fiske et al. (2002) and Leach et al. (2007) on 9-point bipolar scales (end points started with ‘extremely’). A maximum likelihood factor analysis with oblique rotation conducted on these items revealed a clear two-factor solution that accounted for 66.74% of the variance, with warmth and morality items (warm–cold, friendly–unfriendly, helpful–unhelpful, moral–immoral, fair–unfair, loyal–disloyal, trustworthy–untrustworthy, sincere–insincere, well-intentioned–ill-intentioned, and good-natured–bad-natured) loading on the first factor (loadings from 0.531 to 0.998), and competence items (competent-incompetent, skillful-unskillful, capable-incapable, and efficient-inefficient, intelligent-unintelligent, confident-unconfident) loading on the second factor (loadings from 0.519 to 0.882). The scores on these items were averaged to create composite measures of perceived *warmth* (α = 0.93) and *competence* (α = 0.88).

##### Emotional attributions

Participants were asked to indicate how the male target described in the profile should be feeling based on his role within the marriage on 7-point scales ranging from (1) *not at all* to (7) *extremely* on a number of positive and negative moral emotion items. A maximum likelihood factor analysis with oblique rotation conducted on these items revealed a clear two-factor solution that accounted for 61.58% of the variance, with negative moral emotion items (embarrassed, ashamed, humiliated, guilty, angry, and resentful) loading on the first factor (loadings ranged from 0.568 to 0.907), and positive moral emotion items (proud, self-fulfilled, respected, appreciated, satisfied, and gratitude) loading on the second factor (loadings ranged from 0.702 to 0.809). The scores on these items were averaged to create composite measures of *negative emotional attributions* (α = 0.90) and *positive emotional attributions* (α = 0.79).

##### Masculine honor ideals

Participants completed 12 items extracted from the Honor Ideology for Manhood (HIM) scale (Barnes et al., 2012). Barnes et al.’s (2012) HIM is a valid and reliable 16-item scale, developed within the Southern United States context. We decided to leave out four items which included colloquial expressions and one item which seemed unsuitable in the British cultural context. The items excluded were “a real man never lets himself be a ‘door mat’ to other people,” “a real man never leaves a score unsettled,” “a real man can pull himself up by his bootstraps when the going gets tough,” and “a man has the right to act with physical aggression toward another man who trespasses on his personal property.” Using a scale ranging from 1 (*strongly disagree*) to 9 (*strongly agree*), participants rated their level of agreement on six statements tapping into the characteristics of what should define a “real men” (e.g., “A real man is seen as tough in the eyes of his peers”) and six statements tapping into men’s right to demonstrate physical aggression for personal and reputational defense (e.g., “A man has the right to act with physical aggression toward another man who calls him an insulting name”) (α = 0.95). Because these items are phrased ideologically, the HIM scale allowed us to measure both men’s and women’s adherence to masculine honor ideals.

We measured HIM after the manipulations and dependent variables to avoid influencing participants’ evaluations of the targets. Our decision risked that participants’ HIM scores could be affected by the manipulations, but this was not a concern, since participants’ HIM scores did not significantly differ as a function of target (father vs. mother), *F* < 1, role *F*(1,150) = 1.30, *p* = 0.20. Men (*M* = 4.95, *SD* = 1.80) endorsed higher masculine honor ideals than did women (*M* = 3.37, *SD* = 1.64), *F*(1,150) = 5.69, *p* < 0.001, *d* = 0.92.

### Results

Table 1 presents the bivariate correlations, and Table 2 presents the means and standard deviations by participant gender and target role.

**TABLE 1:** Study 1: Bivariate correlations of the study variables.

	**1.**	**2.**	**3.**	**4.**	**5.**
1. Warmth	−	0.74^∗∗^	–0.32^∗∗^	0.45^∗∗^	0.06
2. Competence		−	–0.29^∗∗^	0.37^∗∗^	0.11
3. Positive emotions			−	−0.17^*^	0.12
4. Negative emotions				−	0.01
5. HIM					−

HIM = Masculine honor ideals (measured by 12 items selected from Honor Ideology for Manhood scale; Barnes et al., 2012). Study N = 155. ^*^p < 0.05, ^∗∗^p < 0.01.

**TABLE 2:** Study 1: Means and standard deviations by participant gender and target role on trait judgments and emotional attributions.

	**Caregiver father**	**Breadwinner father**
	***M (SD)***	***M (SD)***
**Men**		
Warmth	7.44 (1.28)^a^	6.00 (1.20)^b^
Competence	7.13 (1.46)	6.87 (1.34)
Positive emotions	4.87 (1.48)	4.50 (0.77)
Negative emotions	2.60 (1.47)	2.93 (1.17)
**Women**		
Warmth	7.34 (1.50)^a^	6.46 (1.29)^b^
Competence	7.36 (1.26)	7.21 (1.53)
Positive emotions	4.60 (1.22)	4.47 (1.10)
Negative emotions	2.82 (1.31)	2.69 (1.02)
Cell size	*n* = 76	*n* = 78

*Character traits were measured in 9-point bipolar scales (1 = extremely cold/incompetent, 5 = neutral, 9 = extremely warm/competent). Positive and negative emotions were measured on 7-point scales (1 = not at all, 4 = somewhat, 7 = extremely). ^*a,b*^Means differ significantly at *p* < 0.01.*

#### Moderation by Masculine Honor Ideals and Participant Gender

To examine the moderating role of masculine honor ideals (HIM) and participant gender on perceived warmth and competence and emotional attributions to the caregiver versus breadwinner fathers, we conducted a set of moderation analyses using the PROCESS macro (Model 3; Hayes, 2018) by mean-centering the predictors for the computation of the interaction terms. We calculated bias-corrected 95% bootstrap confidence intervals for the conditional effects (10,000 bootstrap samples). Table 3 presents the model summary and the conditional effects for male and female participants at low and high levels of HIM.

**TABLE 3:** Study 1: Model summary for the association between Target Role, HIM, Target Role × HIM interaction and outcome variables, and the conditional effects for male and female participants at low and high levels of HIM.

	**Male participants**											
	**Perceived warmth**			**Perceived competence**			**Positive emotions**			**Negative emotions**		
	**Coeff.**	***SE***	**95% CI**	**Coeff.**	***SE***	**95% CI**	**Coeff.**	***SE***	**95% CI**	**Coeff.**	***SE***	**95% CI**
**Predictors**												
TR	–0.60^∗∗∗^	0.12	−0.84, −0.36	–0.17	0.13	−0.42, 0.08	–0.12	0.11	−0.34, 0.09	0.14	0.11	−0.08, 0.36
HIM	0.11^†^	0.07	−0.01, 0.24	0.15^*^	0.07	0.02, 0.29	–0.009	0.06	−0.12, 0.11	0.07	0.06	−0.05, 0.19
TR × HIM	–0.009	0.07	−0.14, 0.12	0.07	0.07	−0.07, 0.20	–0.04	0.06	−0.15, 0.08	–0.07	0.06	−0.19, 0.05
PG	–0.34	0.24	−0.82, 0.13	−0.52^*^	0.25	−1.02, −0.02	0.16	0.22	−0.27, 0.59	–0.05	0.22	−0.49, 0.39
TR × PG	–0.25	0.24	−0.73, 0.23	–0.17	0.25	−0.67, 0.33	–0.05	0.22	−0.48, 0.38	0.37	0.22	−0.07, 0.81
HIM × PG	0.01	0.13	−0.25, 0.27	0.06	0.14	−0.21, 0.33	–0.02	0.12	−0.25, 0.21	–0.12	0.12	−0.36, 0.12
TR × HIM × PG	0.03	0.13	−0.23, 0.28	0.10	0.14	−0.17, 0.37	–0.007	0.12	−0.24, 0.22	−0.24^†^	0.12	−0.47, 0.001
**Conditional TR × HIM effects**												
Male participants	β = 0.005, *F*(1,144) = 0.003			β = 0.12, *F*(1,144) = 1.59			β = −0.04, *F*(1,144) = 0.23			β = −0.20^*^, *F*(1,144) = 5.39		
Female participants	β = −0.02, *F*(1,144) = 0.05			β = 0.02, *F*(1,144) = 0.04			β = −0.03, *F*(1,144) = 0.15			β = 0.04, *F*(1,144) = 0.22		
**Conditional effects**												
Male participants												
Low HIM	−0.75^*^	0.30	−1.34, −0.16	–0.49	0.31	−1.10, 0.13	–0.08	0.27	−0.61, 0.45	0.70^*^	0.27	0.16, 1.24
High HIM	–0.73^∗∗∗^	0.18	−1.09, −0.37	–0.03	0.19	−0.41, 0.35	–0.23	0.16	−0.55, 0.10	–0.04	0.17	−0.37, 0.30
Female participants												
Low HIM	−0.44^*^	0.18	−0.81, −0.08	–0.13	0.19	−0.51, 0.25	–0.04	0.16	−0.36, 0.28	–0.11	0.17	−0.44, 0.22
High HIM	−0.53^†^	0.29	−1.09, 0.04	–0.05	0.30	−0.64, 0.54	–0.16	0.25	−0.66, 0.34	0.04	0.26	−0.48, 0.56

TR = target role (caregiver = −1, breadwinner = 1); HIM = masculine honor ideals; PG = participant gender; Low HIM = Mean − 1SD; High HIM = Mean + 1SD. ^†^p < 0.10, ^*^p < 0.05, ^∗∗^p < 0.01, ^∗∗∗^p < 0.001.

##### Trait judgments

We predicted that caregiver fathers would be perceived as warmer, but not more competent than breadwinner fathers (Hypothesis 1). We did not have directional predictions regarding the moderating role of HIM on perceived warmth and competence, but we still examined the two- and three-way interaction effects for exploratory purposes.

As expected, caregiver fathers were perceived as warmer, but equally competent as the breadwinner fathers. Thus, Hypothesis 1 was supported. No significant Target Role × HIM × Gender interaction effect emerged on perceived warmth or competence. The conditional Target Role × HIM interaction effects for men and for women were also non-significant. These findings indicate that masculine honor ideals did not moderate men’s or women’s perceived warmth and competence of the caregiver (vs. breadwinner) fathers.

##### Emotional attributions

We hypothesized an interaction of target role and HIM such that participants’ (especially men’s) endorsement of HIM would increase their tendency to attribute less positive and more negative emotions to the primary caregiver fathers (Hypothesis 2). We did not predict a main effect of target role, but we still examined this for exploratory purposes.

**FIGURE 2:**
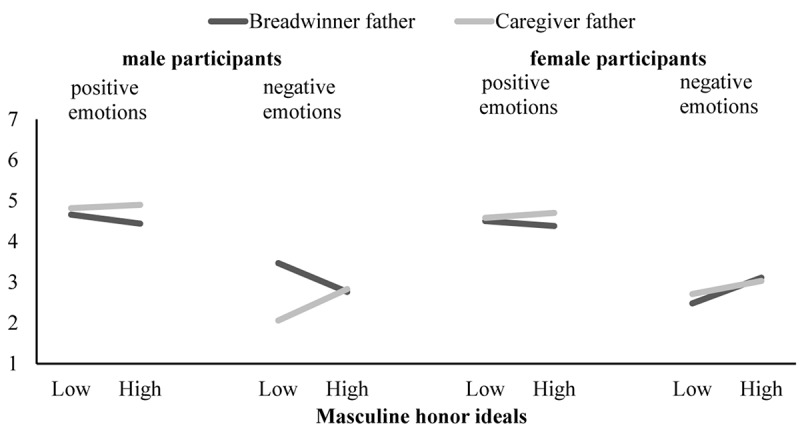
Study 1: Simple slopes for men and women who endorse high levels (*M* + 1*SD*) and low levels (*M* – 1*SD*) of masculine honor ideals on positive emotional attributions and negative emotional attributions to the breadwinner versus caregiver fathers. Simple slope for *low* masculine honor-oriented *male participants* on negative emotions and *caregiver father* on *male participants’* negative emotions were (marginally) significant. All other simple slopes were non-significant.

Participants did not differ in their positive and negative emotional attributions to caregiver versus breadwinner fathers. No Target Role × HIM × Gender interaction effect emerged on positive emotional attributions. The conditional Target Role × HIM interaction effects for men and women were non-significant. But, as expected, Target Role × HIM × Gender interaction effect was significant on negative emotional attributions. Conditional Target Role × HIM interaction was significant for men, but not for women. As expected, men attributed marginally more negative emotions to the caregiver (vs. breadwinner) father as they endorsed higher masculine honor ideals (see Figure 2). These results provided some support for Hypothesis 2 with regards to our prediction on negative emotional attributions.

### Discussion

In line with previous studies (e.g., Gaunt, 2013; Vandello et al., 2013), Study 1 results showed that both men and women perceived the caregiver fathers as warmer than breadwinner fathers. But, caregiver fathers were not perceived less competent than breadwinner fathers. This may be due to continuously changing gender roles in general and the move toward judging targets in non-traditional roles as no less competent than those in traditional roles (see e.g., Pew Research Center, 2008, 2015). Furthermore, warmth and competence judgments of men in caregiver and breadwinner roles were not contingent upon individuals’ endorsement of masculine honor ideals.

With regards to emotional attributions, neither men nor women attributed less positive or more negative emotions to the primary caregiver than breadwinner fathers. This means that our participants did not display negative evaluative judgments of men in a caregiver role relative to men in a breadwinner roles. Nevertheless, as we expected, these emotional attributions were contingent upon men’s (but not women’s) endorsement of masculine honor ideals: higher endorsement of masculine honor ideals was related to men’s tendency to attribute more negative emotions (e.g., shame, humiliation, and resentment) to caregiver fathers. Although, this association was only marginally significant, overall Study 1 provided some evidence that high and low masculine honor-oriented men may be perceiving caregiver fathers differentially in ways that reflect their own social/moral standards. Furthermore, the moderating role of masculine honor ideals was unique for men, implying that masculine reputation concerns may be a potential underlying motive of men’s negative emotional attributions to caregiver fathers.

## Study 2

Study 2 aimed to replicate Study 1 findings using a design with several modifications and minor changes in measures. First, because Study 1 demonstrated that masculine honor ideals moderated men’s negative emotional attributions to caregiver fathers, but not women’s, in Study 2 we only examined evaluations by men. Second, instead of asking men to attribute emotions to the targets described in the profiles, we asked men to imagine themselves as if they were the primary caregiver or the primary breadwinner target described in the profiles, and to report how they would feel about taking on such a role within their own marriage. This was done to more directly capture men’s self-evaluations and internalized standards. In Study 2, we also tested Hypotheses 3, 4a, and 4b and to this end, we asked participants (after they imagined themselves as the breadwinner or the caregiver father) to report how their male friends (status relevant observers), as well as wife and children (status-irrelevant observers) would perceive them, and how they would feel around them.

### Methods

#### Participants

Inputting the smallest effect size from Study 1 (β = 0.24) into G^*^Power determined a sample size of 131 at 0.80 power for Target Role × Masculine honor ideals interaction effect. A new sample of 143 men from the United Kingdom, recruited through Prolific Academic (a United Kingdom-based crowdsourcing for scientific research, see Peer et al., 2017), completed an online survey advertised as “a study on impression formation of married couples.” Participants were a mixture of university students and working adults. Excluding 14 participants who were non-heterosexual^3^, and further 10 participants who failed to pass simple attention checks left data from 119 men (*M*_age_ = 36.55, *SD* = 11.47; 92% White-British). Study 2 sample characteristics were similar to those of Study 1.^4^ Because our final sample size was lower than what was determined *a priori*, we conducted *post hoc* power analysis to ensure that our sample had sufficient power to detect the effect sizes we obtained. G^*^Power indicated that our sample size of 119 would have 0.95 power to detect the smallest effect size (β = 0.29) obtained from our significant Target Role × Masculine honor ideals interaction effects.

#### Design and Procedure

The study used a single factor design with *target role* (breadwinner vs. caregiver) as the between-subjects factor and *masculine honor ideals* measured as a continuous moderator. We used the same profiles as in Study 1, and asked participants to imagine themselves in the described roles and complete the measures listed below.

#### Measures

##### Trait judgments

Same trait judgment dimensions (warmth and competence) were used as in Study 1, except that the measurement scale changed from a 9-point bipolar scale to an explicitly 7-point unipolar scales ranging from (1) *not at all* to (7) *very much*. This was done to communicate to participants only one category (e.g., honesty), rather than two categories (e.g., honesty and dishonesty), which is thought to be cognitively easier for participants to respond (Gannon and Ostrom, 1996). A maximum likelihood factor analysis with oblique rotation conducted on these items revealed a clear two-factor solution that accounted for 69.80% of the variance, with warmth and morality items (warm, friendly, helpful, moral, fair, and loyal) loading on the first factor (loadings from 0.662 to 0.957), and competence items (competent, skillful, capable, and efficient) loading on the second factor (loadings from 0.648 to 0.861). The scores on these items were averaged to create composite measures of perceived *warmth* (α = 0.93) and *competence* (α = 0.88).

##### Emotional attributions

Participants rated how they would feel if they were in the position of the target on 7-point scales ranging from (1) *not at all* to (7) *extremely*. The same items were used to measure emotional attributions as in Study 1, but two extra negative emotion items (*annoyed* and *uncomfortable*) were included. A maximum likelihood factor analysis with oblique rotation conducted on these items revealed a clear two-factor solution that accounted for 52.05% of the variance, with negative moral emotion items (annoyed, resentful, ashamed, angry, humiliated, uncomfortable, and guilty) loading on the first factor (loadings ranged from 0.602 to 0.853), and positive moral emotion items (proud, self-fulfilled, satisfied, gratitude, and appreciated) loading on the second factor (loadings ranged from 0.490 to 0.745). The scores on these items were averaged to create composite measures of *negative emotional attributions* (α = 0.90) and *positive emotional attributions* (α = 0.79).

##### Perceptions/feelings attributed to one’s wife, children, and male friends

Participants were asked to imagine themselves as the male target, and rated how much they think their wife would admire them (e.g., “how appreciative would your wife be of you?”), and find them attractive (e.g., “how attractive would your wife find you?”), how much their children (when they grow up) would admire them (e.g., “how much would the children admire their father?”), how much their male friends would admire them (e.g., “how impressed would your male friends be of you?”, “how much would your male friends pity you? [reverse-coded]), and how dominant/high status they would feel among their male friends (e.g., “how dominant would you feel among your male friends?”). Ratings were given on 7-point scales ranging from (1) *not at all* to (7) *very much*. A maximum likelihood factor analysis with oblique rotation conducted on these items revealed a clear five-factor solution that accounted for 73.99% of the variance, with each conceptually-relevant item loading together under a single factor. Thus, we created composite measures of perceived *admiration of wife* (4 items; α = 0.90; item loadings ranged from 0.538 to 0.897), *attraction of wife* (4 items; α = 0.97; loadings from −0.868 to −0.944)*, admiration of children* (4 items; α = 0.96; loadings from 0.811 to 0.938), *admiration of male friends* (6 items; α = 0.86; loadings from 0.489 to 0.848), *dominance/status among male friends* (3 items; α = 0.87; loadings from −0.572 to −0.952).

##### Masculine honor ideals

As in Study 1, the same 12 items taken from the HIM scale (Barnes et al., 2012) were used to measure participants’ endorsement of masculine honor ideals (α = 0.92). Again, we measured HIM after the profiles and dependent variables to avoid influencing participants’ evaluations of the targets. Our decision risked that participants’ HIM scores could be affected by the manipulations, but this was not a concern, since HIM scores did not significantly differ as a function of Target Role (breadwinner vs. caregiver), *F* < 1.

### Results

Table 4 presents the bivariate correlations, and Table 5 presents the means and standard deviations by target role.

**TABLE 4:** Study 2: Bivariate correlations of the study variables.

	**1.**	**2.**	**3.**	**4.**	**5.**	**6.**	**7.**	**8.**	**9.**	**10.**
1. Warmth	−	0.52^∗∗^	0.36^∗∗^	–0.14	0.59^∗∗^	0.30^∗∗^	0.52^∗∗^	−0.20^*^	–0.11	0.22^*^
2. Competence		−	0.40^∗∗^	−0.19^*^	0.51^∗∗^	0.34^∗∗^	0.43^∗∗^	0.17	0.12	0.26^∗∗^
3. Positive emotions			−	–0.50^∗∗^	0.57^∗∗^	0.46^∗∗^	0.46^∗∗^	0.40^∗∗^	0.24^∗∗^	0.02
4. Negative emotions				−	–0.34^∗∗^	–0.46^∗∗^	–0.36^∗∗^	–0.40^∗∗^	−0.20^*^	0.16
5. Admiration of the wife					−	0.61^∗∗^	0.69^∗∗^	0.17	0.21^*^	0.20^*^
6. Attraction of the wife						−	0.53^∗∗^	0.35^∗∗^	0.32^∗∗^	0.10
7. Admiration of children							−	0.11	0.14	0.17
8. Admiration of male friends								−	0.54^∗∗^	–0.07
9. Dominance/status among male friends									−	0.03
10. HIM										−

HIM = Masculine honor ideals (measured by 12 items selected from Honor Ideology for Manhood scale; Barnes et al., 2012). Study N = 119. ^*^p < 0.05, ^∗∗^p < 0.01.

**TABLE 5:** Study 2: Means and standard deviations by target role on men’s trait judgments, emotional attributions, and perceptions/feelings attributed to their wife, children, and male friends.

	**Caregiver father**	**Breadwinner father**
	***M (SD)***	***M (SD)***
Warmth	$5.77\left(0.79\right){}^{a}$	$4.43\left(1.00\right){}^{b}$
Competence	5.63 (0.82)	5.33 (1.11)
Positive emotions	4.73 (1.11)	4.59 (1.04)
Negative emotions	2.17 (1.28)	2.26 (1.06)
Admiration of wife	$5.53\left(0.99\right){}^{c}$	$4.87\left(1.26\right){}^{d}$
Attraction of wife	4.65 (1.57)	4.59 (1.22)
Admiration of children	$5.75\left(1.19\right){}^{c}$	$5.06\left(1.33\right){}^{d}$
Admiration of male friends	$3.98\left(1.33\right){}^{a}$	$5.03\left(0.94\right){}^{b}$
Dominance/status among male friends	$3.38\left(1.32\right){}^{c}$	$4.05\left(1.26\right){}^{d}$
Cell size	*n* = 55	*n* = 64

**All variables were measured on 7-point scales (1 = not at all, 4 = somewhat, 7 = very much). ^*a,b*^Means differ significantly at p < 0.001. ^*c,d*^Means differ significantly at p < 0.01*.*

#### Moderation by Masculine Honor Ideals

To examine the moderating role of masculine honor ideals (HIM) on men’s social judgments of caregiver versus breadwinner fathers, we conducted a set of moderation analyses on each outcome variable using the PROCESS macro (Model 1; Hayes, 2018) by mean-centering the predictors for the computation of the interaction term. We calculated bias-corrected 95% bootstrap confidence intervals for the conditional effects (10,000 bootstrap samples). Table 6 presents the model summary and the conditional effects of target role on the outcome variables at low and high levels of HIM.

**TABLE 6:** Study 2: Model summary for the association between Target Role, HIM, Target Role × HIM interaction and outcome variables, and the conditional effects of Target Role on the outcome variables at low and high levels of HIM.

	**Perceived warmth**			**Perceived competence**			**Positive emotions**			**Negative emotions**					
	**Coeff.**	***SE***	**95% CI**	**Coeff.**	***SE***	**95% CI**	**Coeff.**	***SE***	**95% CI**	**Coeff.**	***SE***	**95% CI**			
**Predictors**															
TR	–0.67^∗∗∗^	0.08	−0.83, −0.51	−0.15^†^	0.09	−0.33, 0.02	–0.06	0.10	−0.25, 0.13	0.08	0.10	−0.12, 0.27			
HIM	0.17^∗∗^	0.06	0.06, 0.28	0.17^∗∗^	0.06	0.05, 0.29	–0.007	0.07	−0.14, 0.12	0.15^*^	0.07	0.01, 0.28			
TR × HIM	0.05	0.06	−0.06, 0.16	0.07	0.06	−0.05, 0.19	0.29^∗∗∗^	0.07	0.16, 0.42	–0.29^∗∗∗^	0.07	−0.43, −0.15			
**Conditional effects**															
Low HIM	–0.74^∗∗∗^	0.11	−0.97, −0.51	−0.26^*^	0.12	−0.51, −0.02	–0.49^∗∗^	0.14	−0.75, −0.22	0.50^∗∗∗^	0.14	0.22, 0.78			
High HIM	–0.60^∗∗∗^	0.12	−0.83, −0.37	–0.04	0.13	−0.29, 0.20	0.36^∗∗∗^	0.14	0.09, 0.63	−0.35^*^	0.14	−0.63, −0.07			

	**Admiration of wife**			**Admiration of children**			**Attraction of wife**			**Admiration of male friends**			**Dominance/status among male friends**		

**Predictors**															
TR	–0.33^∗∗^	0.10	−0.53, −0.13	−0.35^*^	0.11	−0.57, −0.12	–0.03	0.13	−0.28, 0.22	0.53^∗∗∗^	0.10	0.33, 0.72	0.33^∗∗^	0.12	0.10, 0.56
HIM	0.15^*^	0.07	0.02, 0.29	0.15^†^	0.08	−0.006, 0.30	0.08	0.09	−0.09, 0.25	–0.09	0.07	−0.22, 0.05	0.004	0.08	−0.14, 0.56
TR × HIM	0.19^∗∗^	0.07	0.05, 0.33	0.14^†^	0.08	−0.01, 0.30	0.19^*^	0.09	0.02, 0.36	0.29^∗∗∗^	0.07	0.16, 0.42	0.23^∗∗^	0.08	0.07, 0.39
**Conditional effects**															
Low HIM	–0.61^∗∗∗^	0.14	−0.89, −0.33	–0.56^∗∗∗^	0.16	−0.88, −0.24	−0.31^†^	0.18	−0.66, 0.05	0.10	0.14	−0.18, 0.37	–0.004	0.16	−0.33, 0.32
High HIM	–0.05	0.14	−0.34, 0.23	–0.14	0.16	−0.46, 0.18	0.25	0.18	−0.11, 0.60	0.95^∗∗∗^	0.14	0.68, 1.23	0.67^∗∗∗^	0.16	0.34, 1.00

TR = target role (caregiver = −1, breadwinner = 1); HIM = masculine honor ideals; Low HIM = Mean − 1SD; High HIM = Mean + 1SD. ^†^p < 0.10, ^*^p < 0.05, ^∗∗^p < 0.01, ^∗∗∗^p < 0.001.

##### Trait judgments

We predicted that caregiver fathers would be perceived as warmer, but not more competent than breadwinner fathers (Hypothesis 1). We did not have directional predictions regarding the moderating role of HIM on perceptions of warmth and competence of the targets, but we still examined the two-way interaction effects.

Replicating Study 1, the caregiver fathers were perceived as warmer, but equally competent as the breadwinner fathers. As in Study 1, we did not find a significant Target Role × HIM interaction effect on perceived warmth or perceived competence. This means that men’s judgments of caregiver and breadwinner fathers on warmth and competence were not moderated by masculine honor endorsement.

##### Emotional attributions

We hypothesized an interaction of target role and HIM such that participants’ (especially men’s) endorsement of HIM would increase their tendency to attribute less positive and more negative emotions to being a primary caregiver father (Hypothesis 2). We did not predict a main effect of target role, but we still examined this for exploratory purposes.

**FIGURE 3:**
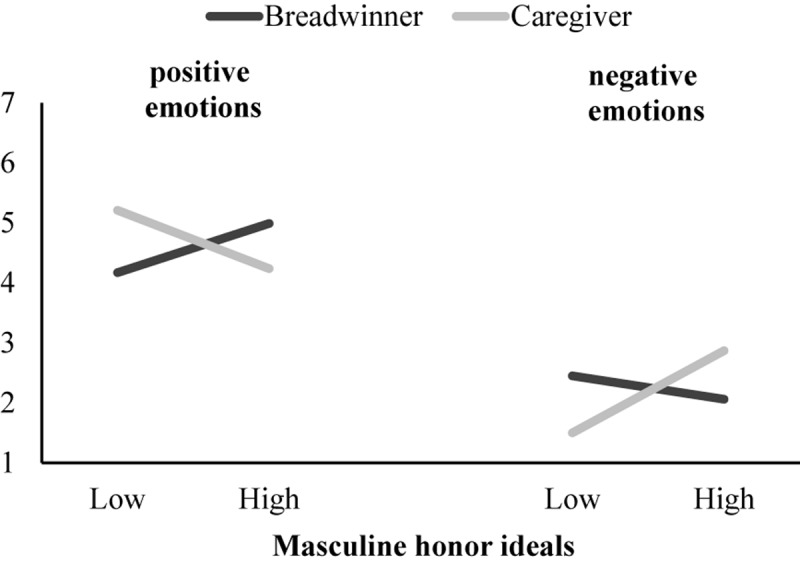
Study 2: Simple slopes for men who endorse high levels (*M* + 1*SD*) and low levels (*M* – 1*SD*) of masculine honor ideals on positive emotional attributions and negative emotional attributions when they imaged being a breadwinner versus a caregiver father. All simple slopes were significant, except the slope for the breadwinner father on negative emotions.

Replicating Study 1, men did not differ in their positive and negative emotional attributions to caregiver versus breadwinner fathers. As in Study 1, Target Role × HIM interaction was significant on negative emotional attributions. A significant Target Role × HIM interaction also emerged on positive emotional attributions. As expected, men attributed less positive and more negative emotions to becoming a caregiver (vs. breadwinner) as they endorsed higher masculine honor ideals (see Figure 3). These results fully supported Hypothesis 2.

##### Perceptions/feelings attributed to wife, children, and male friends

Men perceived that their wife and children would admire them more if they were the caregiver than if they were the breadwinner, but they perceived that their male friends would admire them less, and that they would feel less dominant/high status among male friends if they were the caregiver than if they were the breadwinner. Men did not think that their wife’s attraction to them would differ if they were the caregiver or the breadwinner. These findings suggested that overall men think that becoming a caregiver would be negatively perceived among observers who are status competitors (male friends), but not among those who are not status competitors (one’s wife and children).

We predicted that men’s endorsement of masculine honor ideals would moderate their perceived admiration from family and perceived reputation loss among male friends if they were a primary caregiver (vs. breadwinner) (Hypothesis 3). As predicted, Target Role × HIM interaction was significant on perceived admiration of one’s wife, wife’s attraction, admiration of male friends, dominance/high status among male friends, and marginally significant on admiration of children, providing support for Hypothesis 3.

**FIGURE 4:**
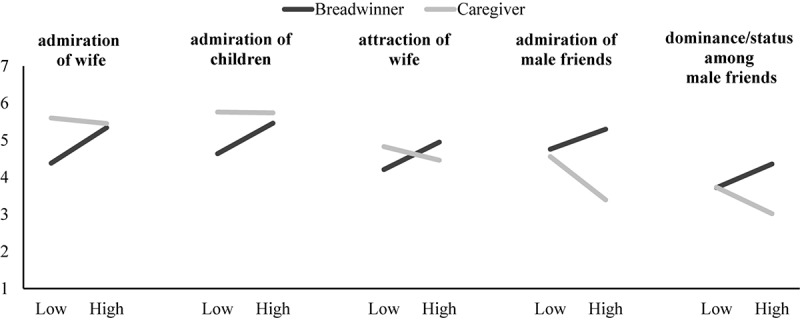
Simple slopes for men who endorse high levels (*M* + 1*SD*) and low levels (*M* – 1*SD*) of masculine honor ideals on perceptions/feelings attributed to wife, children, and male friends when they imaged being a breadwinner versus a caregiver father. Simple slopes for *low* masculine honor-oriented men and *breadwinner father* were significant on admiration of wife and admiration of children. Simple slope for *breadwinner father* was significant on attraction of wife. Simple slopes for *high* masculine honor-oriented men, *caregiver father*, and *breadwinner father* were significant on admiration of male friends and dominance/high status among male friends. All other simple slopes were non-significant.

We found the direction of associations to be consistent with the individual-difference perspective on masculine honor. For high masculine honor-oriented men, imagining oneself as a caregiver (vs. breadwinner) decreased perception that male friends would admire them, and feelings of dominance/high status among male friends, but this was not the case for low masculine honor-oriented men. In contrast, for low masculine honor-oriented men, imagining oneself as a breadwinner (vs. caregiver) decreased perception that one’s wife and children would admire them, and one’s wife would be attracted to them, but not for high masculine honor-oriented men (see Figure 4).

#### Mediated Moderation Analyses

Next, we tested whether high masculine honor-oriented men’s less positive and more negative emotional attributions to becoming a caregiver (vs. breadwinner) is mediated by their perceived loss of reputation among male friends (Hypothesis 4a), and whether low masculine honor-oriented men’s more positive and less negative emotional attributions to becoming a caregiver (vs. breadwinner) is mediated by their perceived gain of their wife’s and children’s admiration (Hypothesis 4b). To do so, first, we created measures for *perceived reputation among male friends* and *perceived admiration of wife and children* as a composite of items measuring perceptions attributed when considering status-relevant others (i.e., male friends; α = 0.88), and perceptions attributed when considering status-non-relevant others (i.e., wife and children; α = 0.94).^5^ These two measures were used as mediators in the mediated moderation model. The correlation between the two mediators was significant but weak (*r* = 0.184, *p* = 0.045), and multicollinearity was not a concern (VIF = 1.000).

**FIGURE 5:**
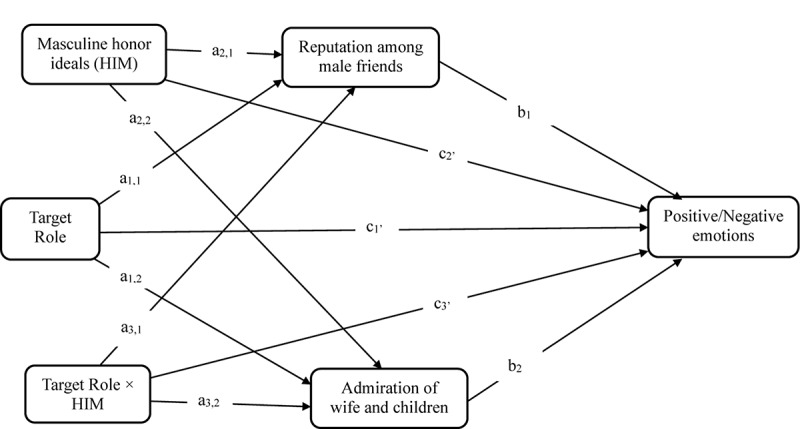
Statistical diagram of the effect of Target Role (caregiver vs. breadwinner), Masculine honor ideals (HIM), and Target Role × HIM interaction on men’s positive and negative emotional reactions mediated by perceived reputation among male friends and perceived admiration of wife and children.

Mediated moderation analysis (also known as conditional indirect process modeling), was conducted using the PROCESS macro (Model 8; Hayes, 2018) by entering the two mediators (perceived reputation among male friends and perceived admiration of wife and children) simultaneously, which allowed us to test Hypotheses 4a and 4b at the same time. Two mediated moderation models were tested: one for positive emotional attributions and one for negative emotional attributions as the outcome variables (the statistical diagram of the mediated moderation model is shown in Figure 5). We calculated bias-corrected 95% bootstrap confidence intervals (CIs) for direct and indirect effects (10,000 bootstrap samples). Table 7A presents all tests of direct and indirect effects, and Table 7B presents the tests of conditional direct and indirect effects. We discuss results here that focus on the pivotal Target Role × HIM interaction effects on positive and negative emotional attributions via the two mediators: perceived reputation among male friends and perceived admiration of wife and children.

**TABLE 7A:** Tests of direct and indirect effects of the mediated moderation model presented in Figure 5.

**Direct effects**	**Path**	**Coeff.**	***SE***	**95% CI**
TR ⇒ Reputation among male friends	*a*_1,1_	0.46^∗∗∗^	0.09	0.29, 0.64
TR ⇒ Admiration of wife and children	*a*_1,2_	–0.34^∗∗∗^	0.10	−0.53, −0.15
HIM ⇒ Reputation among male friends	*a*_2,1_	–0.06	0.06	−0.18, 0.07
HIM ⇒ Admiration of wife and children	*a*_2,2_	0.15^*^	0.07	0.02, 0.28
TR × HIM ⇒ Reputation among male friends	*a*_3,1_	0.27^∗∗∗^	0.06	0.15, 0.39
TR × HIM ⇒ Admiration of wife and children	*a*_3,2_	0.17^*^	0.07	0.03, 0.30
**Outcome: Positive emotions**				
Reputation among male friends ⇒ Positive emotions	*b*_1_	0.22^*^	0.09	0.05, 0.39
Admiration of wife and children ⇒ Positive emotions	*b*_2_	0.47^∗∗∗^	0.08	0.32, 0.63
TR ⇒ Positive emotions	*c*_1__^´_	–0.001	0.10	−0.19, 0.19
HIM ⇒ Positive emotions	*c*_2__^´_	–0.07	0.06	−0.18, 0.04
TR × HIM ⇒ Positive emotions	*c*_3__^´_	0.15^*^	0.06	0.03, 0.27
**Outcome: Negative emotions**				
Reputation among male friends ⇒ Negative emotions	*b*_1_	–0.28^∗∗^	0.10	−0.47, −0.08
Admiration of wife and children ⇒ Negative emotions	*b*_2_	–0.32^∗∗∗^	0.09	−0.50, −0.14
TR ⇒ Negative emotions	*c*_1__^´_	0.09	0.11	−0.12, 0.31
HIM ⇒ Negative emotions	*c*_2__^´_	0.18^∗∗^	0.06	0.05, 0.31
TR × HIM ⇒ Negative emotions	*c*_3__^´_	−0.16^*^	0.07	−0.30, −0.03

**Indirect effects**	**Path**	**Coeff.**	***SE***	**95% CI**

**Outcome: Positive emotions**				
TR ⇒ Positive emotions Via Reputation among male friends	*a*_1,1_^*^ *b*_1_	0.14	0.06	0.04, 0.26
TR ⇒ Positive emotions Via Admiration of wife and children	*a*_1,2_^*^ *b*_2_	–0.16	0.06	−0.29, −0.06
HIM ⇒ Positive emotions Via Reputation among male friends	*a*_2,1_^*^ *b*_1_	–0.008	0.02	−0.06, 0.03
HIM ⇒ Positive emotions Via Admiration of wife and children	*a*_2,2_^*^ *b*_2_	0.08	0.04	0.006, 0.17
TR × HIM ⇒ Positive emotions Via Reputation among male friends	*a*_3,1_^*^ *b*_1_	0.06	0.03	0.01, 0.12
TR × HIM ⇒ Positive emotions Via Admiration of wife and children	*a*_3,2_^*^ *b*_2_	0.08	0.04	0.02, 0.16
**Outcome: Negative emotions**				
TR ⇒ Negative emotions Via Reputation among male friends	*a*_1,1_^*^ *b*_1_	–0.18	0.07	−0.33, −0.07
TR ⇒ Negative emotions Via Admiration of wife and children	*a*_1,2_^*^ *b*_2_	0.09	0.04	0.02, 0.19
HIM ⇒ Negative emotions Via Reputation among male friends	*a*_2,1_^*^ *b*_1_	0.008	−0.02	−0.03, 0.06
HIM ⇒ Negative emotions Via Admiration of wife and children	*a*_2,2_^*^ *b*_2_	–0.06	0.03	−0.13, −0.003
TR × HIM ⇒ Negative emotions Via Reputation among male friends	*a*_3,1_^*^ *b*_1_	–0.07	0.03	−0.14, −0.02
TR × HIM ⇒ Negative emotions Via Admiration of wife and children	*a*_3,2_^*^ *b*_2_	–0.05	0.03	−0.11, −0.01

TR = target role (caregiver = −1, breadwinner = 1); HIM = masculine honor ideals. ^*^p < 0.05, ^∗∗^p < 0.01, ^∗∗∗^p < 0.001.

**TABLE 7B:** Tests of conditional direct and indirect effects of the mediated moderation model presented in Figure 5.

**Conditional direct effects**	**Coeff.**	***SE***	**95% CI**
TR ⇒ Reputation among male friends			
*Low HIM*	0.07	0.13	−0.19, 0.32
*High HIM*	0.86^∗∗∗^	0.13	0.61, 1.11
TR ⇒ Admiration of wife and children			
*Low HIM*	–0.58^∗∗∗^	0.14	−0.86, −0.31
*High HIM*	–0.10	0.14	−0.37, −0.18
TR ⇒ Positive emotions			
*Low HIM*	–0.22	0.12	−0.46, 0.02
*High HIM*	0.22	0.14	−0.05, 0.49
TR ⇒ Negative emotions			
*Low HIM*	0.33^*^	0.14	0.06, 0.61
*High HIM*	–0.14	0.16	−0.46, 0.17

**Conditional indirect effects**	**Coeff.**	***SE***	**95% CI**

**Outcome: Positive emotions**			
TR ⇒ Positive emotions Via Reputation among male friends			
*Low HIM*	0.01	0.03	−0.04, 0.08
*High HIM*	0.19	0.09	0.03, 0.38
TR ⇒ Positive emotions Via Admiration of wife and children			
*Low HIM*	–0.28	0.09	−0.48, −0.11
*High HIM*	–0.05	0.06	−0.17, 0.07
**Outcome: Negative emotions**			
TR ⇒ Negative emotions Via Reputation among male friends			
*Low HIM*	–0.02	0.04	−0.11, 0.05
*High HIM*	–0.24	0.09	−0.42, −0.08
TR ⇒ Negative emotions Via Admiration of wife and children			
*Low HIM*	0.19	0.07	0.06, 0.34
*High HIM*	0.03	0.05	−0.05, 0.13

TR = target role (caregiver = −1, breadwinner = 1); HIM = masculine honor ideals; Low HIM = Mean − 1SD; High HIM = Mean + 1SD. ^*^p < 0.05, ^∗∗^p < 0.01, ^∗∗∗^p < 0.001.

First, we examined the direct effects. The Target Role × HIM interaction predicted higher perceived reputation among male friends (above and beyond Target Role and HIM; path *a*_3,1_). Perceived reputation among male friends, in turn, predicted higher positive emotional attributions and lower negative emotional attributions (above and beyond Target Role, HIM, and their interaction; paths *b*_1_). Similarly, the Target Role × HIM interaction predicted higher perceived admiration of wife and children (above and beyond Target Role and HIM; path *a*_3,2_). Perceived admiration of wife and children, in turn, predicted higher positive emotional attributions and lower negative emotional attributions (above and beyond Target Role, HIM, and their interaction; paths *b*_2_). The Target Role × HIM interaction also directly predicted higher positive and lower negative emotional attributions (above and beyond Target Role, HIM, reputation among male friends and admiration of wife and children; paths *c*_3__^´_). These tests of direct effects provide initial evidence for the predicted Target Role × HIM interaction effect on men’s positive and negative emotional attributions via perceived reputation among male friends and perceived admiration of wife and children.

Next, we examined the indirect effects. The Target Role × HIM interaction effect on positive and negative emotional attributions were mediated by both perceived reputation among male friends and perceived admiration of wife and children. As Table 7B shows, high masculine honor-oriented men’s less positive and more negative emotional attributions to becoming a caregiver (vs. breadwinner) was mediated by perceived loss of reputation among male friends, but perceived loss of reputation among male friends did not mediate low masculine honor-oriented men’s emotional attributions. In contrast, low masculine honor-oriented men’s more positive and less negative emotional attributions to becoming a caregiver (vs. breadwinner) was mediated by perceived loss of admiration of wife and children, but perceived loss of admiration of wife and children did not mediate high masculine honor-oriented men’s emotional attributions. These findings gave support for Hypotheses 4a and 4b.

### Discussion

The findings from Study 2 on trait judgments were consistent with those of Study 1, showing that men perceived a primary caregiver father as warmer, but not less competent than his breadwinner counterpart. These trait judgments were not moderated by men’s endorsement of masculine honor ideals.

Men did not differ in their emotional attributions to the idea of serving as a primary caregiver versus a breadwinner father. This means that overall men do not have negative evaluative judgments of caregiving compared to breadwinning. Nevertheless, these emotional attributions were contingent upon men’s endorsement of masculine honor ideals such that men’s endorsement of masculine honor ideals increased their tendency to react with less positive emotions (e.g., proud, satisfied, and self-fulfilled) and more negative emotions (e.g., ashamed, humiliated, and uncomfortable) to becoming a caregiver (vs. breadwinner) father.

The mediation results were consistent with our hypothesis derived from masculine honor as an individual difference perspective (e.g., Barnes et al., 2012; Saucier and McManus, 2014), which showed that high masculine honor-oriented men’s less positive and more negative emotional reactions to becoming a caregiver (vs. breadwinner) were driven by perceived loss of reputation among their male friends, but not by losing their wife’s and children’s admiration. In contrast, low masculine honor-oriented men’s more positive and less negative emotional reactions to becoming a caregiver (vs. breadwinner) was driven by perceived gain of their wife’s and children’s admiration, but not by perceived loss of reputation among male friends. Together, these findings demonstrated that concern with losing reputation among status-relevant observers (male friends) may manifest as negative feelings regarding taking on childcare tasks, but these concerns seem to be only present among men who are sensitive to maintaining their masculine reputation.

## General Discussion

Across two studies conducted with British participants, we examined how men and women today perceive male targets in caregiver (vs. breadwinner) roles on the fundamental dimensions of social judgment (warmth and competence), and how they emotionally respond to caregiver (vs. breadwinner) fathers. We also examined whether these perceptions and emotional responses are contingent upon endorsement of masculine honor ideals, and investigated the role of potential mediators in these relationships.

Results showed that both men and women perceived the caregiver fathers as warmer, and not less competent than breadwinner fathers. With regards to emotional attributions, neither men nor women differed in their negative or positive emotional attributions to caregiver versus breadwinner fathers. Men’s emotional attributions did not differ when they imagined serving as a primary caregiver versus a primary breadwinner father themselves. Nevertheless, men’s (but not women’s) emotional attributions were contingent upon their endorsement of masculine honor ideals: men’s endorsement of masculine honor ideals increased their tendency to attribute less positive emotions (proud, satisfied, and self-fulfilled) and more negative emotions (ashamed, humiliated, and uncomfortable) to becoming a caregiver (vs. breadwinner) father. As demonstrated by the mediated moderation results in Study 2, high masculine honor-oriented men’s less positive and more negative emotional attributions to becoming a caregiver (vs. breadwinner) were due to perceived loss of reputation among their male friends. In contrast, low masculine honor-oriented men’s more positive and less negative emotional reactions to becoming a caregiver (vs. breadwinner) were due to perceived gain of their wife’s and children’s admiration, but perceived loss of reputation among male friends did not play a role.

Together, these results suggest that high and low masculine honor-oriented men react to becoming a caregiver in ways that reflect their own moral standards and internalized beliefs about the desirable male behavior, which are driven by their differential reputation concerns. Although overall British men currently perceive caregiver fathers as warmer than breadwinner fathers (and no less competent), those who endorse masculine honor ideals think that taking on a full-time caregiver role within their marriage instead of a breadwinner role would make them feel ashamed, humiliated, and resentful, because they would be concerned of what other men would think of them and how this would reflect on their status and prestige in the eyes of their male friends. On the other hand, men who tend to reject masculine honor ideals think that they would feel proud, satisfied, and self-fulfilled in a full-time caregiver role, primarily because they think that their wife and children would admire and appreciate their contribution.

### Theoretical Contributions

The present research contributes to our understanding of why different men may be reacting differently to the idea of becoming a caregiver father in today’s Western societies. The current study found that men’s concern with losing reputation in the eyes of other men manifested as negative emotional reactions to becoming a primary caregiver. However, this relationship was present only for men who were sensitive to maintaining their masculine reputation. Men who were not particularly sensitive to maintaining their reputation in the eyes of other men reacted positively to becoming a primary caregiver. Though results are generally consistent with the precarious manhood theory, they also suggest extensions to this prominent theory, which has been applied to explain various socially destructive behaviors that men engage in (e.g., aggression, social dominance, and risk-taking) (Vandello and Bosson, 2013). According to precarious manhood theory, masculinity is a precarious status which needs constant validation and demonstration. Precarious manhood theorists acknowledge that cultures differ in the degree to which masculine reputation is valued (Bosson and Vandello, 2011), and that “there can be individual differences in the degree to which men personally endorse the notion that manhood is precarious” (Vandello and Bosson, 2013, p. 106). However, these authors note that they have failed to find empirical support for the role of individual differences among men (Vandello and Bosson, 2013). One reason for this may be that these previous studies have focused on men’s gender role conflict, anxiety, and stress as individual difference factors (Eisler and Skidmore, 1987; O’Neil, 2008), but have not measured individual differences in how much men assign importance to maintaining a masculine reputation as we have done in the current study by assessing individual differences in masculine honor ideals. Thus, a more flexible approach – one that considers men’s varying psychology, goals, and motives – may be necessary in examining the outcomes of men’s view of manhood as a precarious identity.

The present research also contributes to the literature on masculine honor from an individual difference perspective and its outcomes, by showing that men who value masculine honor are not limited to protecting their personal reputation through aggressive and confrontational attitudes and behavior as most research to date has shown (e.g., Barnes et al., 2012; Saucier et al., 2016, 2018; O’Dea et al., 2017). Our research connects the literature on masculine honor and research on gender stereotypes, and it is the first to investigate how endorsement of masculine honor ideals, and its underlying reputation concerns, relate to evaluative/affective reactions to targets in counter-stereotypical roles such as men who are primary caregivers.

### Limitations and Future Research Directions

As any other study, the current study is not without limitations. One limitation was that we examined men’s evaluative reactions in terms of their self-conscious emotional reactions to serving as a caregiver or a breadwinner, but did not examine their *intentions* to do so. Although self-conscious emotions construct the link between moral standards and intentions (see Tangney et al., 2007), intention to serve as a caregiver may not always correspond to one’s moral emotions or standards regarding the role of caregiving. Future studies should consider investigating the role of masculine honor ideals and reputation concerns in men’s intention to take on childcare tasks.

Moreover, the current study focused on the *full-time* caregiver model. However, this is by no means the only caregiving arrangement in the modern households. Survey studies show that across the Western world, the dual-earner model is growing with *part-time* caregiving being the most common arrangement in dual-earner households today (Parker and Wang, 2013; Cory and Stirling, 2015). Therefore, men’s feelings and intentions regarding becoming part-time caregivers deserves future research attention. It may be that high honor-endorsing men may not react as negatively to becoming part-time caregivers to their children.

In the current study, high honor-oriented men reported that becoming a caregiver (vs. breadwinner) would have no influence on their wife’s level of appreciation and admiration, whereas low honor-oriented men reported that becoming a caregiver would increase their wife’s appreciation and admiration, which turned out to be the primary driver for low honor-oriented men’s positive feelings about becoming a caregiver. Considering research showing that mothers’ encouragement of paternal participation in childcare influences fathers’ actual participation in childcare (Pleck, 1983; De Luccie, 1995), our results may also imply that high honor-oriented men may not be making inaccurate perceptions of their partners, and that their partners may in fact not be supportive of their involvement with children as caregivers. Previous research has shown that although men want to contribute more to childcare responsibilities, many women discourage their partners’ involvement in child rearing activities because of believing that fathers are unaccustomed and incompetent in performing such tasks (Pleck, 1983; McBride and Rane, 1998). Thus, future research should also examine high and low honor-oriented men’s partners’ attitudes and expectations regarding paternal involvement.

We acknowledge that our data are correlational, and there can be other variables, which may correlate with masculine honor ideals and contribute to men’s negative reactions to caregiving. For instance, masculine honor ideals correlate at least moderately with traditional gender role norms (e.g., Saucier et al., 2016). Traditional gender role norms are generic and cover any social norm regarding how men and women should be and act (e.g., “it sounds worse when a woman swears than when a man does”). However, the underlying psychological concern for all gender role norms may not be a manifestation of men’s concern for upholding masculine reputation in the eyes of other men. Nevertheless, it is possible that endorsing traditional gender role norms can similarly moderate men’s negative emotional reactions to caregiving through concern for masculine reputation, as long as the particular content of these norms are to do with male toughness, aggression, dominance, and status.

Our perspective and findings regarding low honor-oriented men are in parallel with the overall trends in gender norms and expectations shifting toward gender desegregation in work and family roles. Men who are more impervious to threats to their masculine reputation may be responding quicker to these changes by deciding to become more involved in the caregiving of their children, whereas men who are vulnerable to such threats may be more resistant to change (Winegard et al., 2014). Future research should pay more attention to the men who are actively challenging the traditional constructions of manhood, and investigate how individual men react differently to these societal changes in gender norms depending on their motivations.

## Conclusion

Despite the rising number of men and women in counter-stereotypical roles, it is still very rare for men to become primary caregivers within families with dependent children. To gain social psychological insights into men’s lack of interest in childcare tasks, our study examined British men’s and women’s perceptions and emotional responses to caregiver (vs. breadwinner) fathers, men’s emotional responses to the idea of serving as a caregiver (vs. breadwinner) father themselves, as well as the moderating role of masculine honor ideals and potential mediators of these relationships. Results showed that men and women perceived caregiver fathers as warmer (and not less competent) than breadwinner fathers. Yet, higher endorsement of masculine honor ideals was related to men’s greater feelings of shame, humiliation, and resentment about serving as a primary caregiver, and these negative feelings were driven by concern with reputation loss in the eyes of other men. In contrast, lower endorsement of masculine honor ideals was related to men’s greater feelings of pride, satisfaction, and self-fulfillment about serving as a primary caregiver, and these positive feelings were driven by perceived gain of wife’s and children’s admiration and appreciation for them. These findings contribute to the literature on masculine honor from an individual difference perspective, as well as to the broader social science literature by advancing our understanding of why in today’s society some men may be reacting negatively whereas others may be reacting positively to serving as caregiver fathers.

## Ethics Statement

This study was carried out in accordance with the recommendations of the American Psychological Association, with written informed consent from all participants. All participants gave written informed consent in accordance with the Declaration of Helsinki. The protocol was approved by the ethics committee of the School of Psychology at the University of Kent.

## Author Contributions

PG conceived the research idea, designed and conducted the study, collected and analyzed the data, and drafted the manuscript. AU provided critical comments and revisions and approved the manuscript.

## Conflict of Interest Statement

The authors declare that the research was conducted in the absence of any commercial or financial relationships that could be construed as a potential conflict of interest.

## Footnotes

1 All sexual orientations were invited to participate in the survey. However, for the purposes of the current work, non-heterosexuals were excluded from analysis, as the scenarios involved an opposite sex couple. When we included non-heterosexuals and conducted the same analysis, the results did not change.

2 Because participants’ age can influence their gender role attitudes, and judgments of gender role violators (e.g., caregiving men) (e.g., Thornton et al., 1983), we conducted all analysis of Study 1 by controlling for age. All results remained the same.

3 As in Study 1, all sexual orientations were invited to participate in the survey. However, for the purposes of the current work, non-heterosexuals were excluded from analysis, as the scenarios involved an opposite sex couple. When we included non-heterosexuals and conducted the same analysis, the results did not change.

4 Participants’ highest education levels varied (23% high-school, 17% college, 41% undergraduate, and 19% postgraduate) and a majority of them were married or in relationships (51% married, engaged or in a civil union, 25% in a dating relationship, 19% not in a relationship, and 5% divorced). Almost half of the participants (45%) had children. Participants had middle socioeconomic status (*M* = 51.43, *SD* = 19.15 assessed from 0 = low to 100 = high), slightly more liberal political orientation (*M* = 64.60, *SD* = 20.96 assessed from 0 = extremely conservative to 100 = extremely liberal), and they were not religious (*M* = 1.39, *SD* = 0.82 measured from 1 = not at all to 5 = extremely).

5 We combined these measures together to simplify and reduce the number of our mediators in the mediated moderation testing. Our decision to combine the admiration of wife and children items together, and the admiration of male friends and dominance/status among male friends items together was also based on conceptual similarity, such that these items reflect attributions of how one’s family members (status-irrelevant observers) and how one’s male friends (status-relevant observers) perceive them (the participant). Our decision was also statistically justified based on the similar trends in the moderation results reported (see Figure 4) as well as the factor analyses conducted on all of these items. Factor analyses revealed strong factor correlations between the factor comprising the admiration of wife items and the factor comprising the admiration of children items (magnitude of correlation was 0.621), but low correlations with the factors comprising the admiration of male friends items and dominance/status among male friends items (magnitudes were 0.079 and 0.093). Likewise, the two factors comprising the admiration of male friends and dominance/status among male friends had a medium to strong factor correlation (magnitude was 0.485).
